# Supplementary material for: Botulinum Toxin Complex Serotype B-Okra Exerts Systemic Toxicity via the Oral Route by Disrupting the Intestinal Epithelial Barrier
Source: Toxins (Basel). 2025 Sep 4;17(9):443. doi: 10.3390/toxins17090443 (PMC12473987; doi:10.3390/toxins17090443)
Supplement: Supplementary file 1 [file toxins-17-00443-s001.zip › toxins-3775966-supplementary.pdf]

toxins

MDPI

Supplementary Materials: Botulinum Toxin Complex Serotype B-Okra Exerts Systemic Toxicity via the Oral Route by Disrupting the Intestinal Epithelial Barrier

Chiyono Morimoto, Sho Amatsu, Takuhiro Matsumura, Masahiko Zuka and Yukako Fujinaga

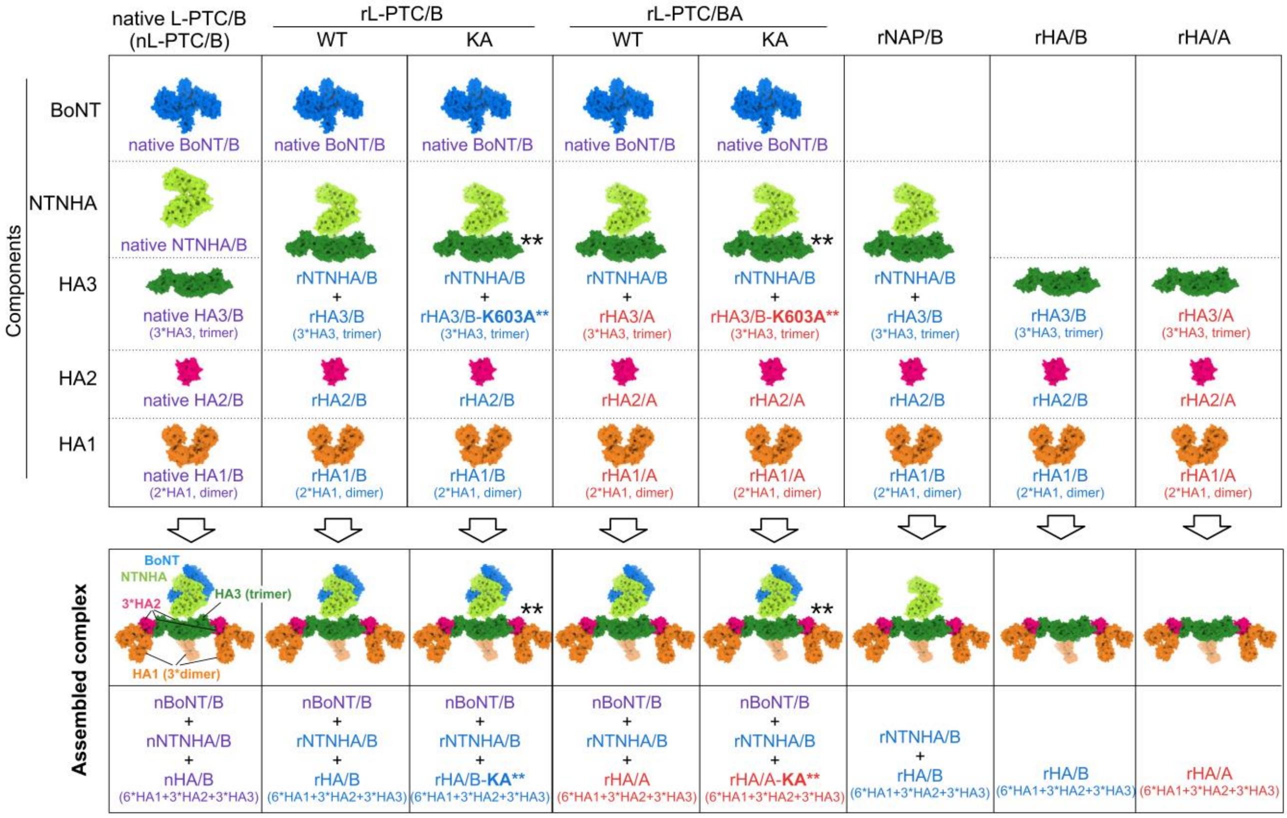

Figure S1. Schematic model of protein preparation. Large progenitor toxin complexes (L-PTCs) are composed of BoNT, NTNHA, HA3, HA2, and HA1 at a molar ratio 1:1:3:3:6. Native L-PTC/B (nL-PTC/B) was produced by *C. botulinum* serotype B strain Okra. Recombinant L-PTCs (rL-PTCs) were reconstituted using native BoNT/B, recombinant NTNHA/B, and recombinant HA/B or HA/A, resulting in rL-PTC/B or rL-PTC/BA, respectively. Neurotoxin-associated protein (NAP), which does not contain BoNT, is composed of NTNHA, HA3, HA2, and HA1 at a molar ratio 1:3:3:6. HA is composed of HA3, HA2, and HA1 at a molar ratio 3:3:6. HA3-K607A mutant is denoted with an asterisk (**).
